# Four-Strand Hamstring Braid Graft for Anterior Cruciate Ligament Reconstruction

**DOI:** 10.1016/j.eats.2025.103623

**Published:** 2025-05-21

**Authors:** Diego Ariel de Lima, Gustavo Vinagre, Gonzalo Samitier, Camilo Partezani Helito, Sergio Marinho de Gusmão Canuto

**Affiliations:** a UFERSA, Universidade Federal Rural do Semi-Árido, Mossoró, Brazil; b Department of Orthopaedic Surgery and Traumatology, Unidade Local de Saúde do Médio Ave, Porto, Portugal; c Hospital Quironsalud de Badalona, Barcelona, Spain; d USP. Grupo de Joelho, Instituto de Ortopedia e Traumatologia, Hospital das Clínicas, HCFMUSP, Faculdade de Medicina da Universidade de São Paulo, São Paulo, Brazil; e Ortoclínica Hospital de Ortopedia, Maceió, Brazil

## Abstract

Graft failure is a significant factor contributing to poor outcomes in anterior cruciate ligament reconstruction. A hamstring graft with a diameter of less than 8 mm is associated with nearly a 7-fold increase in the risk of failure. The technique proposed here introduces a hamstring braid graft configuration for anterior cruciate ligament reconstruction, offering a viable solution in cases where a larger graft diameter is needed. Mastering various hamstring graft preparation techniques is crucial to achieve an appropriate graft diameter and length that aligns with the patient’s anatomy, height, and physical demands. In summary, the present technique, known as the Brazilian Braid, is a 4-strand hamstring braid graft that represents a reliable, easy-to-prepare, and reproducible graft preparation technique.

Anterior cruciate ligament (ACL) injuries are prevalent worldwide, particularly due to sports activities.^1^, ^2^, ^3^, ^4^ Despite recent advances in arthroscopic techniques, devices, and a deeper understanding of knee biomechanics, ACL reconstruction is not always a successful procedure. Approximately 10% to 15% of patients who undergo ACL reconstruction report unsatisfactory outcomes.^5^

Graft failure is one of the primary factors contributing to poor outcomes in ACL reconstruction surgery.^6^ Careful graft selection, based on the individual needs of the patient and therapeutic goals, is crucial to maximizing functional outcomes and patient satisfaction.^7^ The bone–patellar tendon–bone is valued for its strong fixation and bone integration but is associated with a more painful recovery and a higher risk of persistent anterior knee pain.^8^^,^^9^ The quadriceps tendon graft offers robustness and is useful in revision surgeries but may potentially impact quadriceps strength, similar to the rectus femoris graft.^10^^,^^11^ The peroneus longus tendon graft is reserved for specific cases due to its potential impact on ankle function.^12^^,^^13^

Our preference lies with the hamstring tendon grafts (semitendinosus [ST] and gracilis [GT]), which combine strength and flexibility with lower donor site morbidity and a favorable healing rate, despite a potentially longer integration time. However, in cases where the hamstring graft diameter is less than 8 mm, the risk of failure increases nearly 7-fold.^14^

In many cases, the only available grafts are hamstring tendons, and depending on the patient’s body type, the ideal thickness of 8 mm may not be achieved.^6^^,^^14^ Therefore, the proposed technique aims to describe a hamstring braid graft configuration for ACL reconstruction, offering a solution for cases where the graft size or thickness is not achieved using standardized techniques.

## Surgical Technique

The complete technique is demonstrated in Video 1. Pearls and pitfalls are presented in Table 1, and advantages and disadvantages are discussed in Table 2.

**Table 1:** Pearls and Pitfalls

Pearls	1. Imaging examination for surgical planning, especially in revisions. 2. Attention to the positioning of the tendons on the button—the center of the ST and GT should be in the loop of the button. 3. During the making of the braid, it is crucial to keep the graft under tension. If necessary, braid with 2 assistants. 4. Be careful and try to harvest the maximum possible length of the graft to compensate for the shortening that occurs with the braid. 5. It is important to secure the ends of the grafts at the end of the braid with transverse 0 Vicryl sutures to resist unravelling and maintain the intended tension. 6. A critical step is to keep the GT in the center and as symmetrical as possible. The strands of the gracilis tendon are positioned in the center and held together, resulting in the following configuration: 1 strand of ST, 2 strands of GT, and 1 strand of ST. Thus, we end up with 3 fibers for the braid. 7. Use a graft station with measurements and an appropriate tension mechanism to pretension the graft. 8. Clamp the button loop near the gracilis fold with a forceps to prevent the GT from rotating around its own axis during the braiding process.
Pitfalls	1. Special attention should be given to the possible presence of other associated ligament injuries, such as extra-articular ones. 2. In patients with combined ACL and ALL injuries, we prefer the technique of triple-strand braided hamstring graft for the ACL and gracilis strand for the ALL, with a single femoral tunnel.^17^ 3. Place the cortical suspension device before starting the braid configuration. 4. Inadequate continuous tension and angles during the preparation of the braid can lead to a nonuniform final graft configuration. 5. A minimum length of 8 to 9 cm is desirable for secure fixation of the final graft.

ACL, anterior cruciate ligament; ALL, anterolateral ligament; GT, gracilis tendon; ST, semitendinosus tendon.

**Table 2:** Advantages and Disadvantages

Advantages	1. Provides anatomic reconstruction of the ACL. 2. Simple and easy technique that does not require much practice or extensive experience in tissue folding. 3. The braid technique can be used for any ligament reconstruction of the central pivot of the knee, especially the ACL. 4. The Brazilian Braid technique can increase the graft diameter by approximately 1 to 1.5 mm. 5. The braid graft takes on a tape-like shape, mimicking the native form of the ACL and theoretically increasing biomechanical strength. 6. Can be used with fixed or adjustable cortical loop suspension devices. 7. For all ACL reconstructions without associated ALL injury, our preference is the Brazilian Braid.
Disadvantages	1. The braid causes a shortening of approximately 5 to 10 mm in the length of the graft. 2. In some cases, it requires at least 2 operators to braid on an auxiliary table, in order to maintain appropriate tension. 3. Since the braid is made manually, there is no standardization of how tight the braids are.

ACL, anterior cruciate ligament; ALL, anterolateral ligament.

### Surgical Indications

The present technique, known as the Brazilian Braid, can be used for any ligament reconstruction of the central pivot of the knee, such as ACL and posterior cruciate ligament reconstruction.^15^ Special attention should be given to the possible presence of associated ligament lesions, such as extra-articular injuries.

Remarkable signs should be considered to rule out associated anterolateral ligament (ALL) injuries: grade 2 or 3 pivot shift, high-level pivoting sports, ligamentous laxity, and Segond fracture.^16^ In these patients, the combination of an ACL reconstruction with an ALL augmentation can be performed. In those cases, the authors recommend one of the following techniques: (1) triple-strand braided hamstring graft for the ACL and gracilis strand for the ALL, with a single femoral tunnel, or (2) combined ACL and ALL reconstruction using the superficial layer quadriceps tendon graft.^11^^,^^17^

However, in ACL reconstructions without associated ALL injuries, the authors recommend the use of the following graft: the Brazilian Braid.

### Surgical Devices for the Procedure

For femoral fixation, the authors recommend the use of a cortical suspensory adjustable-length device, such as the Endobutton: ACL TightRope (Arthrex), FastFit Button (Razek), GraftMax (ConMed), or Ultrabotton (Smith & Nephew). For tibial fixation, the authors recommend an interference screw.

Another possibility would be the use of 2 interference screws, one for femoral fixation and the other for tibial fixation.

### Necessary Materials for Procedure

The materials needed for the procedure are as follows: 1 cortical suspension fixation device, such as an Endobutton plate; 1 interference screw; 1 cannulated reamer/drill—our preference is an retrograde drill, such as the FlipCutter (Arthrex), Tunneling Drill (Razek), Infinity Knee System (ConMed), or Acufex Trunav Retrograde Drill (Smith & Nephew); a 90° femoral guide (such as the Chambat guide); a 55° tibial guide; 2 guide pins (2 mm); a tendon stripper; and basic materials for arthroscopy. Regarding the cortical suspension fixation device, our preference is an adjustable-loop button model, such as the ACL TightRope (Arthrex), FastFit Button (Razek), GraftMax (ConMed), or Ultrabutton (Smith & Nephew).

### Graft Harvest

An incision of approximately 2 to 3 cm is made over the insertion of the pes anserinus tendons, medial to the tibial tubercle. Subsequently, an oblique incision in the sartorius fascia is made to expose the hamstring tendons. The GT and ST tendons are carefully dissected and released distally at the tibial tubercle. The tendons are then sutured at the ends with Ethibond (Ethicon) thread to facilitate subsequent handling. The GT and ST are dissected and released from fascial adhesions. A tendon stripper is used to release each tendon from its proximal muscular attachment.

### Four-Strand Hamstring Braid Graft Preparation

The harvested autograft is placed on the preparation table, where excess muscle tissue and worn parts of the tendons are removed. The other ends of the tendons are then also sutured with Ethibond thread. Each tendon is folded in half to form 4 strands. After folding the tendons at the midpoint, they are positioned in a cortical suspension fixation device like an Endobutton. Our preference is for adjustable loop button models. If femoral fixation with an interference screw is chosen, instead of folding at the Endobutton loop, each tendon is folded over a high-strength thread, such as FiberWire (Arthrex) or Ethibond.

When folding the tendons, whether at the Endobutton loop or the high-strength thread, care is taken to make the lengths of the strands as similar as possible.

**Fig 1:**
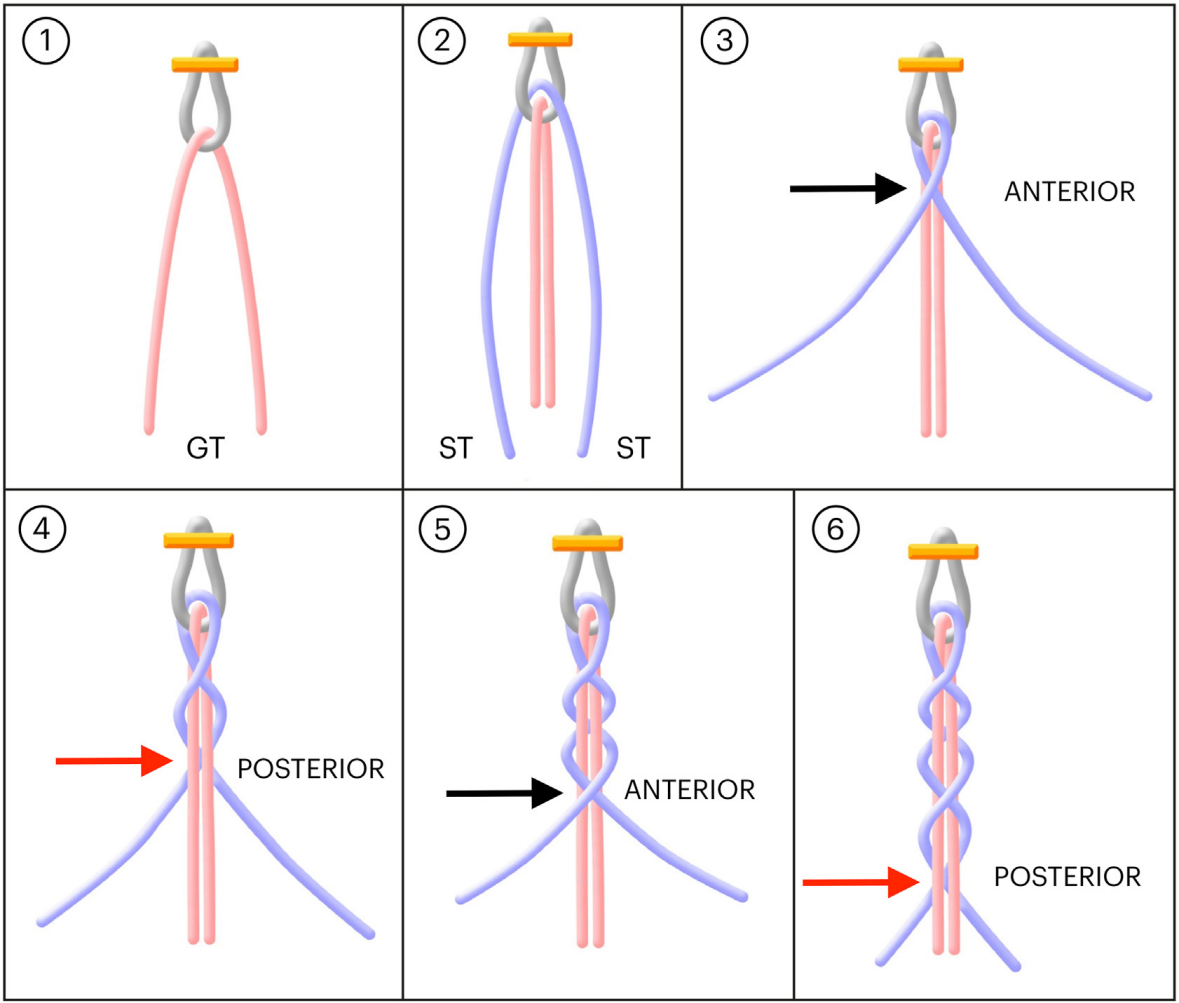
Preparation of the 4-strand hamstring braid graft. (A, B) The gracilis tendon (GT) and semitendinosus tendon (ST) re positioned through the Endobutton loop. The 2 strands of the gracilis tendon are positioned in the center and held together, resulting in the following configuration: 1 strand of ST, 2 strands of GT, and 1 strand of ST. Thus, we end up with 3 fibers for braiding. (C-F) Instead, we keep the GT in the center and braid the ST strands over it. In other words, we perform a concatenated sequence, repeated an integer number of times, crossing the ST strands over the central GT fiber, alternating between “anterior” (black arrow) and “posterior” (red arrow) crossings relative to the GT. These steps are repeated until the end of the graft, and then the fibers are sutured together.

**Fig. 2:**
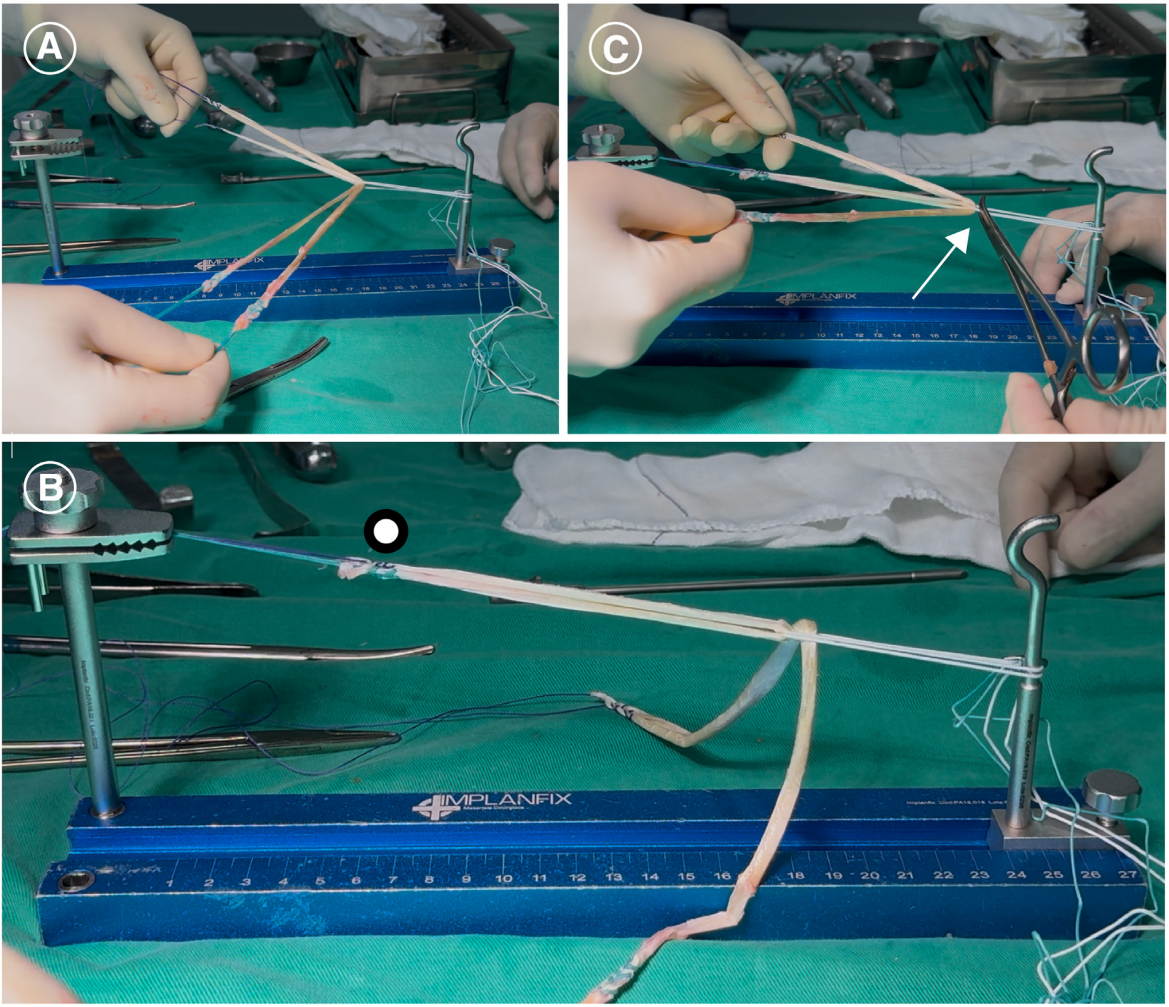
Preparation of the 4-strand hamstring braid graft. (A) The gracilis tendon (GT) and semitendinosus tendon (ST) are positioned through the Endobutton loop. (B) The ends of the GTs (•) are kept under tension and secured on the preparation table. It is crucial to maintain tension during fixation to prevent graft loosening. (C) A useful tip is to clamp the button loop near the gracilis fold with a forceps (white arrow) to prevent the GT from rotating around its own axis during the braiding process.

At this point, one of the main steps in graft preparation is performed. The 2 strands of the gracilis tendon are positioned in the center and held together, resulting in the following configuration: 1 strand of ST, 2 strands of GT, and 1 strand of ST. Thus, we end up with three fibers for braiding (Figs 1 A and B, 2A).

The ends of the gracilis tendons are kept under tension and secured on the preparation table. It is crucial to maintain tension during fixation to prevent graft loosening (Fig 2B). A useful tip is to clamp the button loop near the gracilis fold with a forceps to prevent the GT from rotating around its own axis during the braiding process (Fig 2C).

**Fig 3:**
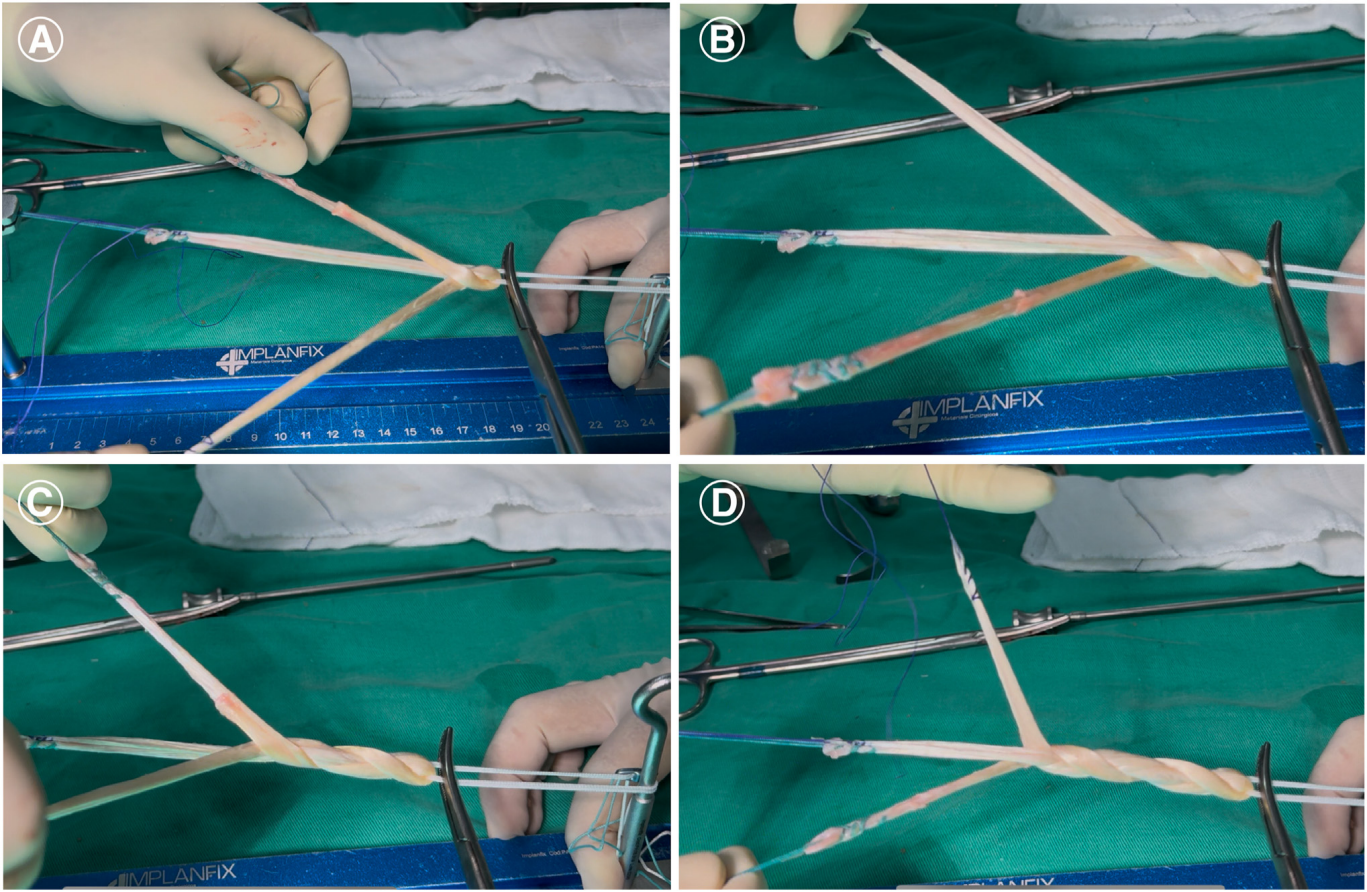
Preparation of the 4-strand hamstring braid graft. (A-D) The gracilis tendon (GT) and semitendinosus tendon (ST) are positioned through the Endobutton. The GT is kept in the center while the ST strands are braided over it. In other words, a concatenated sequence is performed, repeated an integer number of times, with the ST strands crossing over the central GT fiber, alternating between “anterior” and “posterior” crossings relative to the GT. These steps are repeated until the end of the graft, after which the fibers are sutured together

**Fig 4:**
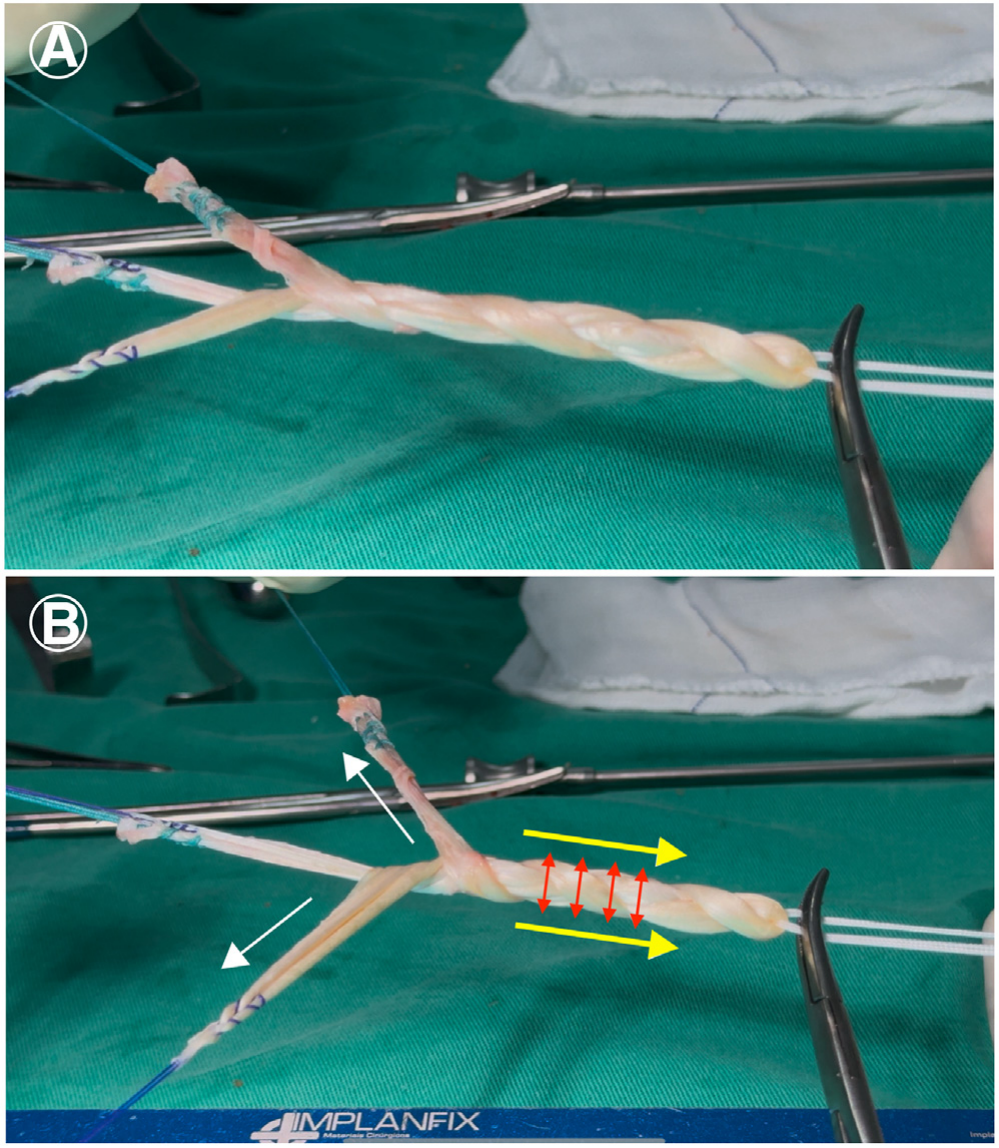
Preparation of the 4-strand hamstring braid graft. (A) This type of braiding allows for simple tensioning of the semitendinosus tendon (ST) fibers over the gracilis tendon (GT) and better control of the desired length of the braided graft. (B) When tensioning the ST strands (white arrow), shortening (yellow arrow) and thickening of the braid (red arrow) occur.

Since the ST tendon is longer than the GT tendon, the GT is kept in the center while the ST strands are braided over it. In other words, a concatenated sequence is performed, repeated an integer number of times, with the ST strands crossing over the central GT fiber, alternating between “anterior” and “posterior” crossings relative to the GT. These steps are repeated until the end of the graft, after which the fibers are sutured together (Fig 3). This type of braiding allows for simple tensioning of the ST fibers over the GT and better control of the desired length of the braided graft (Fig 4).

**Fig 5:**
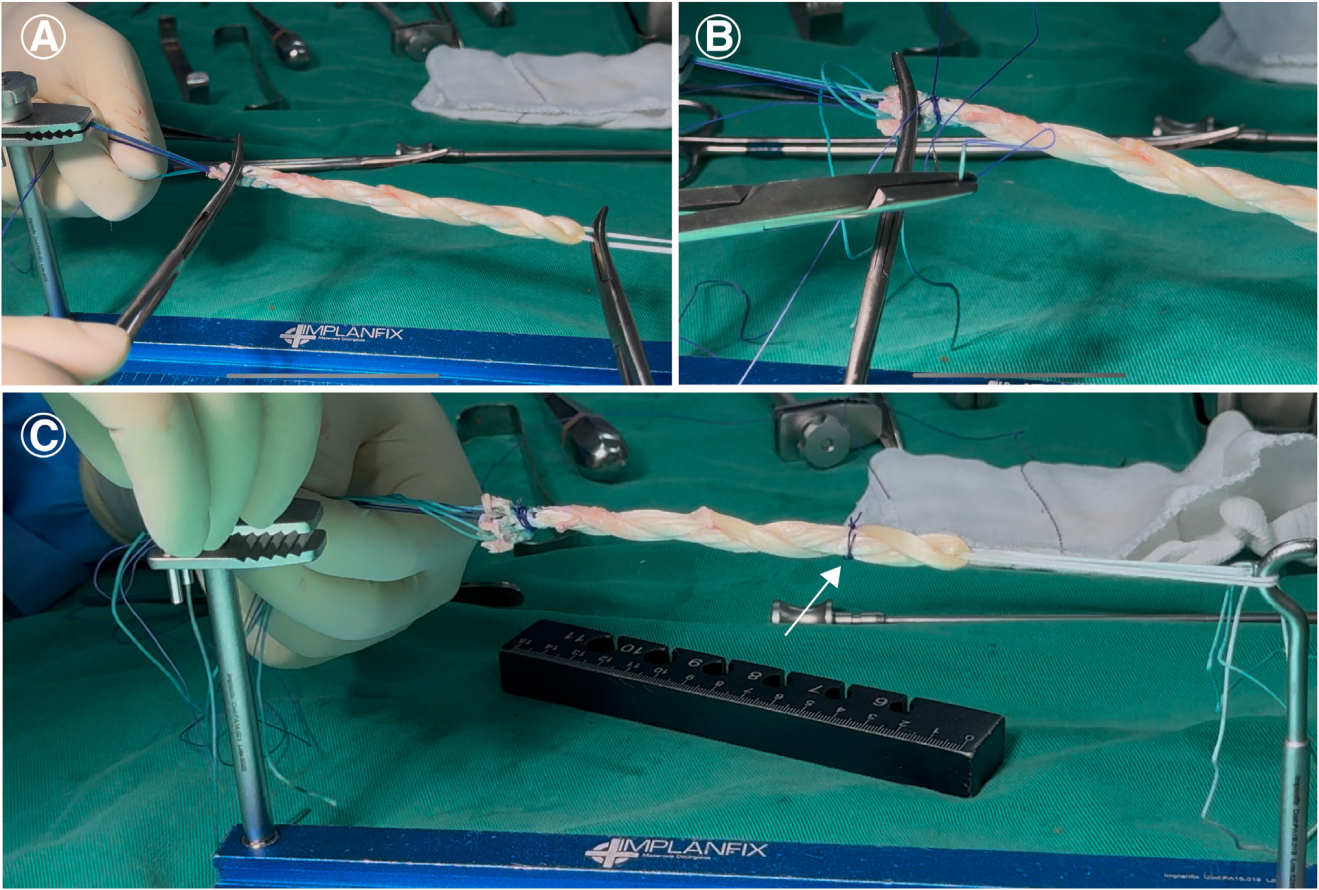
Preparation of the 4-strand hamstring braid graft. (A) It is crucial to keep the braided tendon graft under tension. The firmer the braid, the thicker the graft will be. (B) A 0 Vicryl suture (Ethicon) is performed at the end of the braided graft to secure the braid. (C) A Vicryl suture (white arrow) or a surgical skin marker is used to mark the length of the graft that will be inserted into the femoral tunnel.

A 0 Vicryl suture (Ethicon) is performed at the end of the braided graft to secure the braid (Fig 5). It is crucial to maintain tension throughout the braided tendon graft. The firmer the braid, the thicker the graft will be. The final dimensions of the graft, including length and diameter, are measured. A Vicryl suture or a surgical skin marker is used to mark the length of the graft that will be inserted into the femoral tunnel. After graft preparation, the arthroscopic part of the procedure begins through the anteromedial and anterolateral portals.

### Bone Tunnels and Graft Fixation

The femoral and tibial tunnels can be made according to the surgeon’s preference, taking care to place them as close as possible to the center of the ACL footprint and with a diameter similar to that of the graft.

The authors’ preference is femoral fixation with an Endobutton (facilitates the braid creation) and tibial fixation with an interference screw. If 2 interference screws are chosen, the braid should be made over a high-strength thread, as the graft will be pulled through the femoral tunnel using this suture for subsequent fixation with an interference screw.

## Discussion

An orthopaedic surgeon, particularly a sports knee surgeon, should master more than 1 graft harvesting technique for ACL reconstruction. In this case, the Brazilian Braid, a 4-strand hamstring autograft, can be a useful alternative.

There are numerous studies on graft preparation techniques for ACL reconstruction.^16^, ^17^, ^18^, ^19^ Conte et al.^14^ suggest that grafts smaller than 8 mm in diameter have high failure rates, and according to Figueroa et al.,^20^ increasing the graft diameter by just 0.5 mm can lead to statistically significant increases in the success and longevity of the graft.

This Technical Note demonstrate a 4-strand hamstring autograft braiding technique that increases the graft diameter by nearly 1.0 to 1.5 mm with approximately 10 mm shortening.

Samitier and Vinagre^15^ and Park et al.^21^ have reported a 4-strand braid technique. According to these authors, the 4-strand hamstring autograft braiding can increase the graft diameter by around 1 to 1.5 mm, although with about 5 to 10 mm shortening.

Other theoretical advantages of the 4-strand hamstring autograft braiding technique include obtaining a uniform tape-like graft that seems to reproduce the native shape of the ACL and mimic its mechanical behavior, compensating for the intrinsic viscoelasticity related to soft tissue grafts, and minimizing post-reconstruction elongation that eventually results in laxity and reruptures.^15^^,^^22^ Cavalcante et al.^23^ showed biomechanically that braiding significantly enhances graft strength: a triple-strand braided graft presents axial tensile strength similar to a conventional 4-strand parallel nonbraided graft.

The main limitation of this technique is the graft length, which, after braiding, is shortened by approximately 5 to 10 mm. Thus, in cases of very short grafts, this technique is not recommended.

Therefore, mastering different autologous graft preparation techniques is crucial to obtaining an individualized graft with adequate diameter and length that correspond to the patient’s anatomy, height, and physical demands. The Brazilian Braid technique is a reliable and easy-to-prepare 4-strand hamstring braid autograft configuration, providing a theoretically stronger hamstring graft.

## Disclosures

All authors (D.A.d.L., G.V., G.S., C.P.H., S.M.d.G.C.) declare that they have no known competing financial interests or personal relationships that could have appeared to influence the work reported in this paper.

## Supplementary Data

Video 1 Preparation of the 4-strand hamstring braid graft.

## References

[1] Neufeld E.V., Sgaglione J., Sgaglione N.A. (2025). Anterior cruciate ligament reconstruction graft options. Arthroscopy.

[2] Astur D.C., Xerez M., Rozas J., Debieux P.V., Franciozi C.E., Cohen M. (2016). Anterior cruciate ligament and meniscal injuries in sports: Incidence, time of practice until injury, and limitations caused after trauma. Rev Bras Ortop.

[3] Giugliano D.N., Solomon J.L. (2007). ACL tears in female athletes. Phys Med Rehabil Clin N Am.

[4] Daggett M., Helito C., Cullen M. (2017). The anterolateral ligament: An anatomic study on sex-based differences. Orthop J Sport Med.

[5] Samitier G., Marcano A.I., Alentorn-Geli E., Cugat R., Farmer K.W., Moser M.W. (2015). Failure of anterior cruciate ligament reconstruction. Arch Bone Jt Surg.

[6] Costa G.G., Perelli S., Grassi A., Russo A., Zaffagnini S., Monllau J.C. (2022). Minimizing the risk of graft failure after anterior cruciate ligament reconstruction in athletes. A narrative review of the current evidence. J Exp Orthop.

[7] Carey J.L., Dunn W.R., Dahm D.L., Zeger S.L., Spindler K.P. (2009). A systematic review of anterior cruciate ligament reconstruction with autograft compared with allograft. J Bone Jt Surgery Am Vol.

[8] Pujji O., Keswani N., Collier N., Black M., Doos L. (2017). Evaluating the functional results and complications of autograft vs allograft use for reconstruction of the anterior cruciate ligament: A systematic review. Orthop Rev (Pavia).

[9] Mouarbes D., Menetrey J., Marot V., Courtot L., Berard E., Cavaignac E. (2019). Anterior cruciate ligament reconstruction: A systematic review and meta-analysis of outcomes for quadriceps tendon autograft versus bone–patellar tendon–bone and hamstring-tendon autografts. Am J Sports Med.

[10] Mulford J.S., Hutchinson S.E., Hang J.R. (2013). Outcomes for primary anterior cruciate reconstruction with the quadriceps autograft: A systematic review. Knee Surg Sport Traumatol Arthrosc.

[11] Barroso B.G., Canuto SM. de G., Helito C.P., Rêgo M.C.F., Martins F.S., Rêgo M.C.F. (2024). Combined anterior cruciate ligament and anterolateral ligament reconstruction using the superficial layer quadriceps tendon graft: Surgical technique description. Arthrosc Tech.

[12] He J., Tang Q., Ernst S. (2021). Peroneus longus tendon autograft has functional outcomes comparable to hamstring tendon autograft for anterior cruciate ligament reconstruction: A systematic review and meta-analysis. Knee Surg Sport Traumatol Arthrosc.

[13] Joshi S., Shetty U.C., Salim M.D., Meena N., Kumar S., Rao V.K.V. (2021). Peroneus longus tendon autograft for anterior cruciate ligament reconstruction: A safe and effective alternative in nonathletic patients. Niger J Surg.

[14] Conte E.J., Hyatt A.E., Gatt C.J., Dhawan A. (2014). Hamstring autograft size can be predicted and is a potential risk factor for anterior cruciate ligament reconstruction failure. Arthroscopy.

[15] Samitier G., Vinagre G. (2019). Hamstring braid graft technique for anterior cruciate ligament reconstruction. Arthrosc Tech.

[16] Ariel de Lima D., Helito C.P., Lima F.R.A.D., Leite J.A.D. (2018). Surgical indications for anterior cruciate ligament reconstruction combined with extra-articular lateral tenodesis or anterolateral ligament reconstruction. Rev Bras Ortop (English Ed).

[17] Ariel de Lima D., Helito C.P., de Gusmão Canuto S.M. (2024). Combined reconstruction of the anterior cruciate ligament and anterolateral ligament: Triple-strand braided hamstring graft for the anterior cruciate ligament and gracilis strand for the anterolateral ligament with a single femoral tunnel. Arthrosc Tech.

[18] Jorge P.B., Horita M.M., e Silva M. de O., de Gusmão Canuto S.M., Helito C.P., de Oliveira D.E. (2023). Anterior cruciate and anterior oblique ligament reconstruction using hamstrings and peroneus longus’ anterior half grafts. Arthrosc Tech.

[19] Cerulli G., Placella G., Sebastiani E., Tei M.M., Speziali A., Manfreda F. (2013). ACL reconstruction: Choosing the graft. Joints.

[20] Figueroa F., Figueroa D., Espregueira-Mendes J. (2018). Hamstring autograft size importance in anterior cruciate ligament repair surgery. EFORT Open Rev.

[21] Park H.Y., Gardner B., Kim J.Y. (2021). Four-strand hamstring diamond braid technique for anterior cruciate ligament reconstruction. Arthrosc Tech.

[22] Śmigielski R., Zdanowicz U., Drwięga M., Ciszek B., Ciszkowska-Łysoń B., Siebold R. (2015). Ribbon like appearance of the midsubstance fibres of the anterior cruciate ligament close to its femoral insertion site: A cadaveric study including 111 knees. Knee Surg Sport Traumatol Arthrosc.

[23] Cavalcante M.L.C., Clazzer R., Helito C.P., de Codes R.N., de Lima L.L., de Lima D.A. (2024). Analysis of the biomechanical behavior of an animal model of triple hamstring graft configuration for combined ACL and ALL reconstruction with a single femoral tunnel and a single strand for ALL reconstruction. Rev Bras Ortop.

